# Loop electrosurgical excision procedure combined with cold coagulation for cervical intraepithelial neoplasia and adenocarcinoma in-situ: a feasible treatment with a low risk of residual/recurrent disease

**DOI:** 10.1186/s13027-020-00326-3

**Published:** 2020-10-06

**Authors:** Eun Jung Yang, Nae Ry Kim, Ji Yeon Choi, Wook Youn Kim, Sun Joo Lee

**Affiliations:** 1 Department of Obstetrics and Gynecology, Konkuk University Hospital, Konkuk University School of Medicine, 120-1 Neungdong-ro, Hwayang-dong, Gwangjin-gu, Seoul, 05030 Korea; 2 Department of Pathology, KonKuk University Hospital, Seoul, Republic of Korea

**Keywords:** Cervical intraepithelial neoplasia, Microinvasive carcinoma, Loop electrosurgical excision procedure, Cold coagulation, Resection margin

## Abstract

**Objective:**

This study was performed to evaluate the significance of positive resection margins (RMs) with the loop electrosurgical excision procedure combined with cold coagulation (LEEP with CC) as a definitive treatment for patients with cervical intraepithelial neoplasia (CIN) and adenocarcinoma in-situ.

**Methods:**

We retrospectively reviewed 467 patients who underwent LEEP with CC. A right-angled triangular loop in a single pass followed by a CC (120 °C) to the cone bed for 10 to 20 s was used. Pathology reports and clinical data were obtained and evaluated.

**Results:**

Histopathology evaluation of LEEP tissue samples revealed the presence of CIN 1 in 69, CIN 2/3 in 366, AIS in 5 and invasive carcinoma in 16 (microinvasive squamous cell carcinoma (SCC) and invasive SCC, 13 and 3) patients. Margins were positive in 66 (14.5%) cases: 0 in CIN 1, 54 in CIN 2/3 (12.4%), 1 in AIS (20.0%) and 11 in microinvasive/invasive SCC (68.8%). Although 54 CIN2/3 patients with positive RMs did not undergo additional treatment, 1 of these (1.9%) was confirmed to have residual CIN3 at the first follow-up. Two of 8 (25.0%) microinvasive SCC patients with positive RMs were confirmed to have residual diseases (1 microinvasive SCC and 1 invasive SCC) after hysterectomy. Four out of 360 (1 positive RM, 3 negative RM) CIN cases recurred during the study period.

**Conclusions:**

These results suggest that CIN patients with positive RMs after LEEP with CC may be followed up without additional treatment.

## Introduction

Cervical intraepithelial neoplasia (CIN) has been treated in many different ways including various ablative and excisional procedures [1]. Treating women with CIN reduces the risk of invasive cervical cancer by 95% [2, 3]. The loop electrosurgical excision procedure (LEEP) is a cervical conization procedure using an electrosurgical loop. Since the introduction of LEEP in 1989, it has become one of the most frequently performed gynecological procedures [4, 5].

Previous studies have demonstrated that LEEP is simpler [6] and yields short- and long-term results that are identical to those obtained with more aggressive cold knife conization [6–8]. Although LEEP has a low surgical morbidity [9], some patients experience complications such as postoperative hemorrhaging. To minimize hemorrhagic complications, Allam et al introduced LEEP combined with cold coagulation (LEEP with CC) [10]. This study reported a lower rate of post-treatment bleeding compared with the rates of other LEEP methods. Moreover, the authors suggested that treatment of CIN using LEEP and CC combined could have less abnormal cervical cytologic findings in women who had incomplete excision of their initial lesion compared to LEEP alone. Meanwhile, previous studies reported positive margin rates of 16 to 46.3% after LEEP [7, 11–13]. Although several earlier studies have reported that replicate surgical specimens of patients with involved resection margins (RMs) frequently have no residual neoplasia [14–17], the margin status of conization specimens is considered to be an important predictor of residual neoplasia [18, 19]. Therefore, the American Society for Colposcopy and Cervical Pathology (ASCCP) guidelines recommend “cervical cytology and endocervical curettage at 4-6 months is preferred, but repeat excision or hysterectomy is acceptable” for the cases of “CIN 2, 3 with positive margins” [20].

The present study was designed to evaluate the relation of margin status with residual/recurrent disease in patients treated by LEEP with CC in routine practice. All cases were diagnosed as CIN or adenocarcinoma in-situ (AIS) with colposcopically directed biopsies before LEEP.

## Material and methods

We retrospectively reviewed the medical records and pathology reports of patients who underwent LEEP with CC for the treatment of CIN at Konkuk University Hospital in Seoul, Korea, from August 2005 to December 2018. The inclusion criteria were age ≥ 21 years old, not pregnant, and preoperatively confirmed as having CIN or AIS by colposcopically directed biopsy. Patients with microinvasive or invasive cancer diagnosed before LEEP were excluded from the study. The RM status had been determined on all cases that underwent LEEP with CC. However, cases lost to follow-up after treatment were excluded from the residual/recurrent disease analysis.

Colposcopically directed biopsies were performed on acetowhite areas. Endocervical curettage (ECC) biopsies were performed when transformation zones could not be evaluated by colposcopy. When acetowhite areas were not seen, random cervical punch biopsies in four quadrants together with ECC biopsies were performed. CIN1 cases were recommended for LEEP if they had persistent lesions for more than 2 years or wanted surgery due to concerns regarding cancer within 2 years. Patients with CIN2/3 or AIS underwent LEEP unless they wanted a hysterectomy. When higher grades of CIN, microinvasive and invasive cancer were reported pathologically after LEEP, the diagnoses were upgraded according to the pathology reports. RMs were considered positive in cases where CIN2/3 or more was present on the inked margin [21]. We initially recommended hysterectomy to patients with AIS or microinvasive squamous cell carcinoma (SCC). However, if patients wanted to preserve their fertility, they were followed up without retreatment regardless of the RM status, in accordance with the results of a previous study [14]. Patients with invasive SCC or adenocarcinoma underwent radical hysterectomy or trachelectomy. Residual disease was defined as detection of CIN2/3 or a higher-grade lesion during the first 6 months of follow-up [22]. Recurrence was defined as detection of CIN2/3 or higher-grade lesion thereafter [22]. Patients with residual/recurrent diseases underwent management according to ASCCP guidelines [20, 23].

### Loop electrosurgical excision procedure combined with cold coagulation

LEEP with CC was performed on an in-patient basis using general anesthesia (monitored anesthesia care) by an expert gynecologic oncologist (SJ LEE). LEEP used a right-angled, triangular loop in a single pass with a 3.8-MHz radiosurgery unit (Ellman® International, Inc., NY, USA). A cold coagulator (120 °C) (Eurosurgical®, Guildford, UK) was applied to the cone bed for 10 to 20 s after the excision for hemostasis and destruction of any residual lesion. Before the procedure, 5% acetic acid was applied to the cervix for 1 to 2 min to localize cervical lesions. Hemostatic agents (e.g., Monsel’s solution or fibrin sealant) were not applied after LEEP.

### Follow-up

Negative RM CIN cases were routinely followed up at 4 to 6, and 12 months with pap smear and/or HPV DNA testing. After 12 months, routine annual follow-up was recommended according to guidelines. Positive RM CIN cases were evaluated with pap smear, HPV DNA testing and ECC at 4 to 6 months. In cases where pap smear and HPV DNA testing were negative at 12 months, routine annual follow-up was subsequently pursued.

This study was approved by the institutional review board at Konkuk University Hospital.

### Statistical analysis

Categorical variables were assessed with the χ^2^ test using IBM® SPSS® statistics 22.0 software (IBM SPSS Statistics, Chicago, IL, USA). A *P*-value < 0.05 was considered statistically significant.

## Results

### Clinical data

During the study period, 456 patients underwent LEEP using a right-angled triangular loop with CC. The median age of patients was 38 years (range, 21 to 80 years). After LEEP, 19 of 98 (19.4%) CIN1 cases were upgraded to CIN2/3 according to the pathology report. Forty of 89 (44.9%) CIN2 cases were upgraded according to the pathology report as follows: 38 to CIN3 and 1 each to AIS and to microinvasive SCC. Fifteen of 226 (6.6%) CIN3 cases were upgraded to 12 microinvasive SCC and 3 invasive SCC following LEEP. Seventy-five (16.4%) CIN cases were lost to follow-up after LEEP. All patients who were diagnosed postoperatively as having AIS, microinvasive SCC or invasive SCC were followed up at regular intervals for a median period of 48 months (range, 4 to 157 months) (Table 1). During the study period, 21 patients (5 AIS, 13 microinvasive SCC, 3 invasive SCC) were free of recurrent disease.

**Table 1:** Characteristics of patients who underwent loop electrosurgical excision procedure combined with cold coagulation

Parameter	Results (*n* = 456)
Median age (range) in years	38.0 (21.0–80.0)
Median follow-up period (range) in months	
AIS/Microinvasive SCC/invasive SCC	48.0 (4.0–157.0)
Histologic diagnosis before LEEP, n (%)	
CIN1	98 (21.5)
CIN2/3	128/226 (77.6)
AIS	4 (0.9)
Final histologic diagnosis after LEEP, n (%)	
CIN1	79 (17.3)
CIN2/3	89/267 (78.1)
AIS	5 (1.1)
Microinvasive SCC	13 (2.9)
Invasive SCC	3 (0.7)
Positive resection margin after LEEP, n (%)	66 (14.5)
^a^Endocervical margin	39 (8.6)
Exocervical margin	22 (4.8)
Both margins	5 (1.1)

*AIS* adenocarcinoma in-situ, *SCC* squamous cell carcinoma, *CIN* cervical intraepithelial neoplasia

^a^Endocervical margin; defined as endocervical resection margin and/or deep resection margin

### Resection margins and residual diseases

After LEEP, 66 (14.5%) of 456 patients had positive RMs. Of these, 22 (4.8%) involved the exocervix, 39 (8.6%) the endocervix and 5 (1.1%) both the exocervix and endocervix (Table 1). Endocervical RM was defined here as endocervical RM and/or deep RM. CIN1 cases had no positive RMs, whereas 54 (15.2%) of 356 CIN2/3 cases, 1 (20.0%) of 5 AIS cases, 8 (61.5%) of 13 microinvasive SCC cases, and 3 (100.0%) of 3 invasive SCC cases had positive RMs. Higher grades of disease tended to have higher rates of positive RMs.

Residual disease was analyzed in 381 patients who were followed up continuously in our institute (Tables 2 and 3). One of 44 (2.3%) CIN2/3 patients with positive RMs revealed residual CIN3 at 4 months and underwent LEEP. The remaining 43 patients were followed up without treatment. All patients showed no residual disease during the study period. One AIS patient with positive RM underwent hysterectomy immediately, but no residual lesions were reported after hysterectomy. However, one AIS patient with negative RM revealed residual AIS after hysterectomy. Three other negative RM AIS patients had no residual disease during follow-up. Seven of 8 microinvasive SCC patients with positive RMs underwent hysterectomy within 1 month after LEEP; one patient had microinvasive SCC and another had invasive SCC. One microinvasive SCC case with positive RMs (endocervix and exocervix) was followed up without additional surgical treatment. This patient has shown ‘no evidence of disease’ for 46 months. Five negative RM microinvasive SCC cases (3 underwent hysterectomy, 2 without additional surgical treatment) had no residual disease during the study period. Three invasive SCC patients with positive RMs were diagnosed as having no residual disease after radical surgery.

**Table 2:** Final diagnosis and resection margin status after LEEP, excluding cases lost to follow-up (*n* = 381)

Diagnosis after-LEEP, n	Positive RM, n (%)	Residual disease, n (%)
CIN1, 70	0	0
CIN2/3, 290	44 (15.2)	1 (0.3)
	Endocervix 25^a^	1 (CIN3, LEEP at 4 mo)
	Exocervix 18^a^	0
	Both margins 1^a^	0
AIS, 5	1 (20.0)	0
	Endocervix 1^b^	0
	Exocervix 0	
	Both margins 0	
Microinvasive SCC, 13	8 (61.5)	2 (15.4)
	Endocervix 5	1^b^ (microinvasive SCC)
	Exocervix 0	
	Both margins 3	1^b^ (invasive SCC)
Invasive SCC, 3	3 (100.0)	0
	Endocervix 2^c^	0
	Exocervix 0	
	Both margins 1^c^	0

*LEEP* loop electrosurgical excision procedure, *RM* resection margin, *CIN* cervical intraepithelial neoplasia, *AIS* adenocarcinoma in-situ, *SCC* squamous cell carcinoma

^a^follow-up without additional LEEP (pap smear, HPV DNA testing and endocervical curettage at 4–6 months);

^b^hysterectomy

^c^radical hysterectomy, radical trachelectomy

**Table 3:** Characteristics of patients with positive resection margins after loop electrosurgical excision procedure combined with cold coagulation

		Resection margins			
Age	Diagnosis	endocervix	exocervix	Residual tumor	Patient status
50	AIS	+	–	No^b^	NED, 85 mo
46	Microinvasive SCC	+	+	Invasive SCC^b^	NED, 19 mo
36	Microinvasive SCC	+	–	No^b^	NED, 28 mo
49	Microinvasive SCC	+	–	No^b^	NED, 151 mo
37	Microinvasive SCC	+	+	No^a^	NED, 46 mo
60	Microinvasive SCC	+	–	CIN1^b^	NED, 116 mo
46	Microinvasive SCC	+	+	No^b^	NED, 156 mo
57	Microinvasive SCC	+	–	Microinvasive^b^	NED, 14 mo
40	Microinvasive SCC	+	–	No^b^	NED, 4 mo
56	Invasive SCC	+	+	CIN1^c^	NED, 139 mo
35	Invasive SCC	+	–	No^c^	NED, 48 mo
41	Invasive SCC	+	–	No^c^	NED, 104 mo

*SCC* squamous cell carcinoma, *NED* no evidence of disease

^a^follow-up without additional treatment

^b^hysterectomy

^c^radical hysterectomy, radical trachelectomy

### Recurrent disease during follow-up period

Although there were no positive RM cases in the CIN1 group, one case developed recurrent CIN3 within 12 months (Table 4). In the CIN2/3 group, 3 cases (one positive RM and 2 negative RM) were diagnosed as having recurrent CIN3. When recurrent CIN1 cases were included (CIN1 + CIN2/3), the positive RM group showed a higher rate of recurrence compared to the negative RM group (9.1% vs 4.9%). Nevertheless, there was no statistical significance between these two groups (*P* = 0.298) (Table 4). AIS, microinvasive SCC and invasive SCC cases showed no recurrent disease during the follow-up period.

**Table 4:** Abnormal histology during follow-up period (*n* = 381)

	Abnormal histology during follow-up period		
Diagnosis after-LEEP	Positive RM, (n / %)	Negative RM, (n / %)	*P*-value
CIN1	No case	CIN1^a^ (2 / 2.9)	–
		CIN3^c^ (1 / 1.4)	
CIN2/3	CIN1^a, b^ (3 / 6.8)	CIN1^a, b, c^ (10 / 4.1)	0.298
	CIN3^b^ (1 / 2.3)	CIN3^b, c^ (2 / 0.8)	
AIS	NED	CIN1^b^ (1 / 25.0)	–
Microinvasive SCC	NED	NED	–
Invasive SCC	NED	No cases	–

^a^follow-up without additional treatment

^b^LEEP

^c^hysterectomy

^d^radical hysterectomy, radical

## Discussion

We present here an analysis of 381 women who underwent LEEP using a right-angled triangular loop combined with CC for CIN confirmed preoperatively by colposcopically directed biopsy. This study found a low rate of residual disease (0.8%), despite the fact this procedure showed a positive RM rate of 14.7%. Of note, CIN1/2/3 and AIS cases showed a low rate of only 0.3% (1/365) with residual disease. During the follow-up period, recurrent disease occurred in 1.1% of cases (4/365, 1 with positive RM and 3 with negative RM). Based on these results, we suggest that positive RM cases with a final diagnosis of CIN or AIS and who undergo LEEP with CC can be followed up by pap, HPV DNA testing and ECC without re-excision or hysterectomy. However, the present study indicates that patients diagnosed as having microinvasive SCC with positive RMs following LEEP with CC may have a higher rate of residual disease (15.4%, 2/13) than CIN/ AIS cases. Moreover, 2 cases were reported as invasive SCC and microinvasive SCC after hysterectomy. In spite that 3 microinvasive SCC cases without additional treatment (1 with positive RM, 2 with negative RMs) revealed no residual/recurrent disease during follow-up, we suggest that microinvasive SCC cases with negative RM who undergo LEEP with CC can be followed up without additional surgical treatment.

The present study showed a positive RM frequency of 14.5%, which was similar to previous studies using LEEP. However, residual/recurrent diseases were diagnosed less frequently. Moreover, the current study demonstrated that exocervical or endocervical RMs did not have different effects on the residual/recurrent status. We believe this was due to the use of a right-angled triangular loop in a single pass and the cold coagulator used in our study. Miroshnichenko et al [24] reported that LEEP using a ring-shaped loop was less likely to yield a single intact specimen and that an increase in the number of specimens obtained had a statistically significant negative effect on pathology interpretation. Adequate pathology interpretation using appropriate specimens is required to reduce residual tumors. Matsumura et al [25] previously suggested that one adequate specimen could be obtained in the majority of cases by using a triangular probe and a rigid linear electrode. This is because its relatively large size allows resection of the entire transformation zone and because its linearity allows resection of any lesion extending into the endocervix [25]. Despite having similar positive RM rates, the present study may have more accurate data compared to other previous reports using a ring-shaped loop. Secondly, combined cold coagulator after LEEP might be beneficial both as a hemostatic technique and for reducing the proportion of abnormal smears during follow-up [10]. From the literature, approximately 30 to 50% of cases with involved margins were found to have no residual tumor upon subsequent hysterectomy [17, 26, 27]. This likely indicates the presence of a mechanism that clears residual tumor cells in remnant cervical tissue after conization. Paterson et al [26] and White et al [28] suggested that local activation of cellular immunity after conization causes the regression of residual tumor cells. Additionally, hemostatic measures such as cold coagulation may play a role in destroying residual tumor at the RM, or may influence regression to normal tissue [14].

A strong point of our study was the analysis of all cases that had been treated according to standard practice guidelines at a new hospital since opening. Therefore, we believe the results were not biased due to operator or treatment policy factors. Secondly, the analysis was performed on CIN or AIS cases that were confirmed by colposcopically directed biopsy. Hence, preoperatively selected cases are likely to provide reliability to the study results.

There are however several limitations to this study. Firstly, this was a retrospective case series with a relatively small number of patients. The conclusion of this study was determined by low rate of recurrence. Second, the study did not focus on postoperative complications such as postoperative bleeding, menstruation bleeding volume, pregnancy or preterm delivery. Although a larger volume or destruction by cold coagulator can result in a lower rate of positive RMs, the postoperative complications could increase. However, previous studies using a right-angled triangular loop and/or cold coagulator revealed similar or fewer postoperative complications compared with studies using a ring-shaped loop [14, 25]. Therefore, we believe the rate of postoperative complications in the present study may have been reasonable.

In conclusion, positive RM CIN or AIS cases that undergo LEEP using a right-angled triangular loop combined with CC can be followed up by pap, HPV DNA testing and ECC without re-excision or hysterectomy. However, despite the low rate of residual tumor, re-excision or hysterectomy could be safer for patients diagnosed with positive RM microinvasive SCC or invasive SCC after LEEP. Further large-scale studies will be required to allow more firm conclusions to be drawn.

## Data Availability

All the relevant data for this analysis have been presented in the body of this manuscript and the associated tables. The original data sources and the dataset used in this analysis is available upon reasonable request to the corresponding author..

## Abbreviations

RMs: Resection margins
LEEP: Loop electrosurgical excision procedure
CC: Cold coagulation
CIN: Cervical intraepithelial neoplasia
SCC: Squamous cell carcinoma
ASCCP: American Society for Colposcopy and Cervical Pathology
AIS: Adenocarcinoma in-situ
ECC: Endocervical curettage
